# Early outcomes of component separation techniques: an analysis of the Spanish registry of incisional Hernia (EVEREG)

**DOI:** 10.1007/s10029-021-02449-x

**Published:** 2021-07-02

**Authors:** J. A. Pereira-Rodriguez, A. Bravo-Salva, B. Montcusí-Ventura, P. Hernández-Granados, V. Rodrigues-Gonçalves, M. López-Cano, the EVEREG Registry Members

**Affiliations:** 1 Department of Surgery, Hospital del Mar – Parc de Salut Mar, Passeig Maritim 25-29, 08003 Barcelona, Spain; 2 Department of Experimental and Health Sciences, Universitat Pompeu Fabra, Barcelona, Spain; 3 Department of Surgery, Hospital Universitario Fundación Alcorcón, Madrid, Spain; 4 Abdominal Wall Surgery Unit, Hospital Universitari Vall d’Hebron, Barcelona, Spain; 5 Department of Surgery, Universitat Autònoma de Barcelona, Barcelona, Spain

**Keywords:** Incisional hernia, Component separation technique (CST), Hernia registry, Abdominal wall surgery specialization

## Abstract

**Aim:**

To analyze the outcomes of component separation techniques (CST) to treat incisional hernias (IH) in a large multicenter cohort of patients.

**Methods:**

All IH repair using CST, registered in EVEREG from July 2012 to December 2019, were included. Data on the pre-operative patient characteristics and comorbidities, IH characteristics, surgical technique, complications, and recurrence were collected. Outcomes between anterior (ACS) and posterior component separation (PCS) techniques were compared. Risk factors for complications and recurrences were analyzed.

**Results:**

During the study period, 1536 patients underwent CST (45.5% females) with a median age of 64.0 years and median body mass index (BMI) of 29.7 kg/m^2^. ACS was the most common technique (77.7%). Overall complications were frequent in both ACS and PCS techniques (36.5%), with a higher frequency of wound infection (10.6% vs. 7.0%; *P* = 0.05) and skin necrosis (4.4% vs. 0.1%; *P* < 0.0001) with the ACS technique. Main factors leading to major complications were mesh explant (OR 1.72; *P* = 0.001), previous repair (OR 0.75; *P* = 0.038), morbid obesity (OR 0.67; *P* = 0.015), ASA grade (OR 0.62; *P* < 0.0001), COPD (OR 0.52; *P* < 0.0001), and longitudinal diameter larger than 10 cm (OR 0.58; *P* = 0.001). After a minimum follow-up of 6 months (median 15 months; *N* = 590), 59 (10.0%) recurrences were diagnosed. Operations performed in a non-specialized unit were significantly associated with recurrences (HR 4.903, CI 1.64–14.65; *P* = 0.004).

**Conclusion:**

CST is a complex procedure with a high rate of complications. Both ACS and PCS techniques have similar complication and recurrence rates. Operations performed in a specialized unit have better outcomes.

## Introduction

The component separation technique (CST) has become popular in recent years for incisional hernia (IH) repair [1, 2]. Currently, the following two main CSTs are being used: anterior components separation (ACS) [3] and posterior components separation (PCS) [4]. In both techniques, a mesh is added to reinforce abdominal wall closure and to promote long-term stability [3–5], and both ACS and PCS techniques have proven to be effective [5–7]. Furthermore, in some cases they represent the last chance to achieve a good quality of life for patients who have to modify their lifestyle due to a hernia [8, 9].

However, most of the evidence regarding the use of both CSTs is derived from single experiences or high-volume hospitals [6, 10]. There is a lack of data in a multicenter cohort of patients. Real-world evidence (i.e., registries) can provide information from different institutions on routine practice of CST in unselected patients and can answer questions that are more difficult to analyze with other study designs (i.e., randomized studies). The registry of incisional hernia repair (EVEREG) [11] was started in 2012 and was promoted by the Abdominal Wall Chapter of the Spanish Association of Surgeons (AEC), and it represents one of the few registries of these characteristics present across Europe and the rest of the globe.

The aim of this work is to analyze the outcomes of CST comparing the results of anterior vs. posterior techniques in patients registered in the EVEREG, and to identify the factors predicting complications and recurrences of CST.

## Methods

### Patients

EVEREG is an online database accessible on the internet (http://www.evereg.es/). The register of patients is anonymous, and there are 178 participating centers across the country (38% of 467 National Health System Hospitals). EVEREG is permanently open to all centers that want to participate, and all types of hospitals are included. The registry data structure, committee’s approval process, and data collection system have already been described previously [11]. Briefly, it is a prospective database maintained by the surgeons in charge of each center, in which the parameters of the patients, the type of hernia, operations, and complications are collected for each CST performed. The follow-up is performed through clinical control with an appointment one month, 6 months, 1 year, and 2 years after surgery. Patients undergoing a CST technique (anterior or posterior) and registered in the EVEREG from July 2012 to December 2019 were included and analyzed.

### Variables

Demographic variables, including patient’s age, gender, and body mass index (BMI), obesity, and overweight following WHO classification [14] were collected. Comorbidities selected were hypertension, diabetes mellitus, chronic obstructive pulmonary disease, chronic renal failure, oncologic history, abdominal aortic aneurysm, and American Society of Anesthesiologists’ (ASA) physical status score [12].

IHs were classified according to their location and size according to the EHS classification [15] and complexity [16]. Previous repair and prior use of mesh were identified. Pre-operative optimization with progressive pneumoperitoneum (PPP) or botulinum toxin (BT) was also recorded [17].

Registered surgery characteristics were as follows: clinical setting (i.e., elective or emergency), type of CST (ACS or PCS), concomitant intra-abdominal procedures (i.e., adhesiolysis, bowel resection, and cholecystectomy), panniculectomy, and whether the operation was performed in the Abdominal Wall Unit (AWU). Mesh type, space of mesh placement, and type of fixation were chosen according to the cases and preference of each surgeon.

Perioperative complications, including surgical site occurrences (SSO) (seroma, wound infection, wound hematoma, and skin necrosis), reoperation, and mortality related to surgery during the first postoperative month, were recorded and classified according the Clavien-Dindo grade [18]. After hospital discharge, patients were followed-up at 1 month, 6 months, and one and two years after surgery. Recurrence was evaluated by clinical examination; and in case of doubt, an ultrasound or computed tomography was indicated.

Exclusion criteria were lack of data for the analysis and absence of follow-up data after surgery. For long-term complications, a minimum 6-month follow-up was considered for the analysis.

The development of the study was performed following the international guidelines of clinical investigation (Ethics Code and Helsinki Declaration) and according to the legal regulations for confidentiality and personal data according to Spanish law. The local ethics committee approved the study protocol (2012/4908/I).

### Statistical analysis

The statistical analysis was performed using the IBM statistical package for the Social Sciences (SPSS) program (IBM Inc., Rochester, MN, USA) version 25 for Windows. Quantitative variables were expressed as mean and standard deviation (SD) and qualitative variables as proportions. To analyze the association between qualitative variables, we used the Chi-squared test (*χ*^2^) or Fisher’s exact test when necessary, as well as Student’s t test or the Mann–Whitney *U* test for quantitative variables. The normality of the distribution of the quantitative variables was verified using the Kolmogorov–Smirnov test. Statistical significance was established at *P* < 0.05. The odds ratio (OR) of IH recurrence was calculated with its 95% confidence intervals (CI). In the multivariate analysis for complications, the predictive capacity of each variable and its independence from the other predictor variables were analyzed using a binomial logistic regression model by sequentially introducing the variables with an input F of 0.5. A Cox proportional hazards regression model was used to analyze the risk factors related to recurrence.

## Results

From July 2012 to December 2019, a total of 11,612 IHs were registered in the EVEREG. A total of 1536 (13.3%) patients underwent CST and were available for analysis. A total of 1193 (77.6%) patients were treated with an ACS technique and 343 (22.4%) with a PCS technique. All operations were performed by an open approach.

Table 1 shows the characteristics of the patients and the comparison between both techniques. Males represented 54.5% of the cohort. Median age at time of surgery was 64 years (IQR 55.0–71.0 years), with 31.3% being older than 70 years. Median BMI was 29.7 kg/m^2^ (IQR 26.5–33.5), with a 48.7% rate of obesity (BMI > 30 kg/m^2^). One-third of the cohort (*N* = 502, 32.7%) had an ASA score III–IV with a higher percentage in ACS patients (34%; *P* = 0.05), and arterial hypertension was most frequent in ACS patients (53.5%; *P* = 0.022).

**Table 1:** Pre-operative demographics of patients and hernia characteristics

	*N* = 1536	ACS *N* = 1193	PCS *N* = 343	*P*
Demographics and comorbidities				
Age, years, median (IQR)	64.0 (55.0–71.0)	64.0 (54.0–72.0)	64.0 (55.0–70.0)	0.972
Age > 70 years, *N* (%)	480 (31.3)	383 (32.1)	97 (28.3)	0.178
Sex female, *N* (%)	699 (45.5)	552 (46.3)	147 (43.0)	0.282
BMI, kg/m^2^ median (IQR)	29.7 (26.5–33.5)	29.7 (26.6–33.5)	29.4 (26.3–33.3)	0.399
BMI > 30 kg/m^2^ (obesity), *N* (%)	747 (48.7)	586 (49.2)	161 (46.9)	0.452
BMI > 35 kg/m^2^ (severe obesity), *N* (%)	293 (19.1)	229 (19.2)	64 (18.7)	0.808
Alcohol abuse, *N* (%)	204 (13.3)	159 (13.3)	45 (13.1)	0.920
Smoking *N* (%)	403 (26.2)	324 (27.2)	79 (23.0)	0.126
AHT, *N* (%)	791(51.5)	633 (53.3)	158 (46.1)	0.022
Diabetes mellitus, *N* (%)	336 (21.9)	268 (22.5)	68 (19.8)	0.297
COPD, *N* (%)	277 (18.0)	227 (19.8)	50 (14.6)	0.059
CRF, *N* = 657 (%)	65 (9.9)	30 (8.9)	35 (11.0)	0.368
Immunosuppression *N* = 524 (%)	79 (15.1)	69 (15.8)	10 (11.4)	0.286
Oncologic history, *N* (%)	411 (26.8)	324 (27.2)	87 (25.4)	0.508
AAA, *N* = 524 (%)	8 (1.5)	6 (1.4)	2 (2.3)	0.532
ASA III–IV, *N* (%)	502 (32.7)	405 (34.0)	97 (28.4)	0.050
Hernia characteristics				
Location				
Midline, *N* (%)	1349 (87.8)	1071 (89.8)	278 (81.0)	< 0.0001
Trocar, *N* (%)	55 (3.6)	29 (2.4)	26 (7.6)	< 0.0001
Ostomy, *N* (%)	65 (4.2)	39 (3.3)	26 (7.6)	< 0.0001
Other, *N* (%)	71 (4.6)	67 (5.6)	4 (1.2)	0.001
Defect size*				
Area, cm^2^ (SD)	176,9 (140.7)	187.7 (140.2)	148.7 (138.1)	< 0.0001
Transverse > 10 cm, (W3) *N* (%)	822 (65.9)	642 (71.0)	180 (52.5)	< 0.0001
Longitudinal > 10 cm, *N* (%)	965 (77.4)	748 (82.7)	217 (63.3)	< 0.0001
Previous repair, *N* (%)	486 (31.6)	357 (29.9)	214 (37.6)	0.007
Previous mesh, *N* (%)	452 (93.0)	326 (91.3)	126 (97.7)	0.015
Classification of complexity**				
Minor, *N* (%)	233 (15.2)	175 (14.7)	58 (16.9)	
Moderate, *N* (%)	577 (37.6)	447 (37.5)	130 (37.9)	
Major, *N* (%)	726 (47.2)	571 (47.9)	155 (45.2)	
Pre-operative treatment				
Pneumoperitoneum, *N* (%)	73 (4.8)	60 (5.0)	13 (3.8)	0.342
Botulinum toxin, *N* (%)	78 (5.1)	34 (2.8)	44 (12.8)	< 0.0001

*BMI* body mass index, *AHT* arterial hypertension, *COPD* chronic obstructive pulmonary disease, *CRF* chronic renal failure, *AAA* abdominal aortic aneurysm, *ASA* American Society of Anaesthesiologists physical status classification

*EHS classification; **Slater NJ et al. [16]

The most frequent type of hernia originated in a previous midline laparotomy (87.8%), with 65.9% of W3 class according to the EHS classification [16]. A total of 31% of patients had a previous repair, with mesh in 93.0% of cases, and 47.2% of patients were classified as having major complexity [16]. PPP was used as prior therapy in 73 patients (4.8%) and BT in 78 patients (5.1%). Hernias treated with ACS were larger, most frequently in the midline, and more frequently received PPP. PCS hernias more frequently underwent a previous repair with mesh and a pre-operative therapy with botulinum toxin.

IH characteristics and comparisons are also displayed in Table 1.

Most of the operations were elective (96.9%), with a high frequency of a concurrent procedure, and the most frequent was bowel resection (12%). ACS was the most common technique (77.6%), and 78.8% of the operations were performed at an AWU (Table 2).

**Table 2:** Characteristics of the operations and technique of repair

	*N* = 1536	ACS *N* = 1193	PCS *N* = 343	*P*
Type of procedure				
Elective, *N* (%)	1489 (96.9)	1150 (96.4)	339 (98.8)	0.021
Urgent, *N* (%)	47 (3.1)	43 (2.8)	4 (0.3)	
Operative time, min, median (IQR)	150.0 (90.0–210.0)	135.0 (90.0–200.0)	170.0 (120.0–240.0)	0.032
Length of stay, days, median (IQR)	6.00 (4.00–8.00)	6.00 (4.00–8.00)	6.00 (4.00–8.00)	0.123
AWU*, *N* = 821 (%)	647 (78.8)	363 (74.4)	284 (85.3)	< 0.0001
Associated procedures				
Bowel resection, *N* (%)	176 (11.5)	145 (12.2)	31 (9.0)	0.110
Panniculectomy, *N* (%)	122 (7.9)	90 (7.5)	32 (9.3)	0.281
Adhesiolysis, *N* (%)	53 (3.5)	46 (3.9)	7 (2.0)	0.105
Cholecystectomy, *N* (%)	39 (2.5)	33 (2.8)	6 (1.7)	0.291
Mesh removal, *N* (%)	40 (2.6)	30 (2.5)	10 (2.9)	0.681
Technical details				
Mesh type				
No mesh *N* (%)	8 (0.5)	7 (0.6)	1 (0.3)	0.503
Double mesh *N* (%)	372 (24.3)	226 (19.1)	146 (42.7)	< 0.0001
Reticular *N* (%)	1041 (90.1)	909 (94.7)	132 (67.3)	< 0.0001
Composite *N* (%)	104 (9.0)	45 (4.7)	59 (30.1)	< 0.0001
Biosynthetic *N* (%)	5 (0.4)	1 (0.1)	4 (2.0)	0.027
Biologic *N* (%)	6 (0.5)	5 (0.5)	1 (0.5)	0.985
Mesh area, cm^2^ (SD)	763.4 (653.2)	785.9 (673.3)	556.3 (350.5)	0.001
Mesh position				
Inlay *N* (%)	30 (2.0)	27 (2.3)	3 (0.9)	0.100
Onlay *N* (%)	615 (40.2)	610 (51.4)	5 (1.5)	< 0.0001
Retromuscular and preperitoneal *N* (%)	779 (51.0)	459 (38.7)	320 (93.6)	< 0.0001
Intraperitoneal *N* (%)	94 (6.2)	81 (6.8)	13 (3.8)	0.40
Mesh fixation				
Staples *N* (%)	250 (17.1)	243 (21.7)	7 (2.0)	< 0.0001
Suture *N* (%)	1137 (77.1)	856 (75.6)	281 (81.9)	0.015
Glue *N* (%)	367 (24.2)	312 (26.5)	55 (16.1)	< 0.0001

*AWU abdominal wall unit

Types, position, and fixation of prostheses are shown in Table 2. Only eight patients did not receive a mesh, and 24.3% of cases were repaired using two meshes, more frequently in posterior repair (*P* < 0.0001). When only a mesh was used, the preferred type was reticular polypropylene (90.1%), which was less frequently used in PCS (67.3%). Laminar biosynthetic and biological materials were anecdotally used (0.9%). In ACS, the preferred position was *onlay* (51.2%); and in PCS, the preferred position was *sublay* (93.6%). Mesh fixation with a suture was the more frequently used method (77.1%), and staplers and glue were more frequently used in the ACS technique than in the PCS technique.

Complications, including mortality (1.2%), were frequent after both techniques (36.5%). The ACS technique was related to a significantly higher incidence of wound infection (*P* = 0.05) and skin necrosis (*P* = 0.0001). Comparison by grade did not show any difference. More complex hernias were correlated with an increased percentage of complications (*P* < 0.0001) (Table 3).

**Table 3:** Postoperative 30-day complications

	*N* = 1536	ACS *N* = 1193	PCS *N* = 343	*P*
Overall, *N* (%)	560 (36.5)	421 (35.3)	139 (40.5)	0.076
Intra-operative *N* (%)	45 (2.9)	31 (2.6)	14 (4.1)	0.151
Clavien-Dindo grade				
Grade I	210 (13.7)	137 (11.5)	73 (21.3)	
Grade II	254 (16.5)	207 (17.4)	48 (14.0)	
Grade IIIA	37 (2.4)	33 (2.8)	4 (1.2)	
Grade IIIB	29 (1.9)	24 (2.0)	5 (1.4)	
Grade IV	11 (0.7)	4 (0.3)	7 (2.0)	
Grade V	18 (1.2)	16 (1.3)	2 (0.6)	
SSO, *N* (%)	419 (27.3)	320 (26.8)	99 (28.9)	0.455
Seroma, *N* (%)	228 (14.8)	168 (14.1)	60 (17.5)	0.117
Wound infection, *N* (%)	150 (9.8)	126 (10.6)	24 (7.0)	0.050
Wound haematoma, *N* (%)	94 (6.1)	68 (5.7)	26 (7.6)	0.200
Skin necrosis, *N* (%)	53 (3.5)	52 (4.4)	1 (0.1)	< 0.0001
Reoperation, *N* (%)	41 (2.7)	30 (2.5)	11 (3.2)	0.483
Hernia complexity*				
Minor, *N* (%)	56 (24.0)	39 (22.3)	17 (29.3)	
Moderate, *N* (%)	175 (30.2)	136 (30.4)	39 (30.0)	
Major, *N* (%)	329 (45.3)	246 (43.1)	83 (53.5)	

*Slater NJ et al. [16]

Significant factors leading to any type of postoperative complications in the multivariate analysis are shown in Table 4.

**Table 4:** Multivariate analysis of risk factors for complications

	*P*	OR	95% C.I	
			Low	High
BMI > 35 kg/m^2^	0.015	0.675	0.492	0.927
COPD	0.000	0.527	0.376	0.739
Longitudinal diameter > 10 cm	0.001	0.578	0.413	0.808
Midline	0.005	0.531	0.340	0.827
Parastomal	0.017	0.461	0.244	0.870
Previous repair	0.038	0.754	0.578	0.985
ASA III–IV	0.000	0.617	0.470	0.810
Bowel resection	0.003	0.510	0.326	0.798
Simultaneous hernia repair	0.020	0.460	0.239	0.885
Premuscular repair	0.067	1.320	0.981	1.777
No glue	0.006	0.659	0.489	0.886
Panniculectomy	0.013	0.579	0.375	0.892
Intra-operative complications	0.000	0.138	0.055	0.347

A minimum follow-up of six months (median 14.7 months) was completed in 590 cases (38.4%), and the overall recurrence rate was 10.0% (*N* = 59) without any significant differences on comparing CSTs (ACS 10.8% *vs*. PCS 7.5%; *P* = 0.25). In univariate analysis, immunosuppression (OR = 1.25; CI 0.97–1.61; *P* = 0.008), emergency repair (OR = 1.28; CI 0.94–1.74; *P* = 0.007), suture repair (OR = 10.15; CI 7.95–12.97; *P* = 0.003), premuscular (*onlay*) repair (OR 1.05; CI 0.99–1.12), fixation without staples (OR = 2.35; CI 1.03–5.35; *P* = 0,03), no use of glue (OR = 2.23; CI 1.10–4.70; *P* = 0.02), postoperative complications (OR = 1.98; CI 1.03–1.16; *P* = 0.001), seroma (OR = 1.11; CI 1.01–1.22; *P* = 0.005), SSO (OR = 1.13; CI 1.02–1.16; *P* = 0.004), and operation performed in a non-AWU (OR = 2.05; CI 1.05–4.00; *P* = 0.035) were related to a higher frequency. In the Cox multivariate analysis, non-AWU operation was the only factor significantly associated with recurrences (HR 4.903, CI 1.64–14.65; *P* = 0.004).

## Discussion

To our knowledge, this is one of the largest studies of CST for the treatment of IH. A previous larger study (2245 cases) included all types of ventral hernia (primary and incisional) [19]. The analysis of this series shows that CST is a complex operation, mostly performed in high-risk patients with large major complex hernias, which frequently requires associated procedures and has a high risk of complications. Recurrences are comparable to those with the other techniques [12], but when the operation is performed in an elective setting, with a mesh and by an AWU, the rate of recurrences is lower.

In our series, patients undergoing CST were an aged population with a high proportion of obesity (30.3 kg/m^2^ average BMI) and other comorbidities; one-third of these patients were classified as ASA III or IV, which has been shown to contribute to worse outcomes. Our population was very similar to that in a previous series [10, 20–22].

It is striking that, of the total registered operations, 13.3% needed a CST, while in the largest series, the published rate was 3.3% [19]. Considering the complexity of cases, 34% of the hernias had a transverse diameter less than 10 cm, reaching 48% in the PCS cases; thus, the area of the defects was less than that reported in other studies carried out in specialized centers [9]. However, when we analyzed the hernias in relation to their complexity classification [16], 85% of the patients had criteria of moderate or major complexity. This led us to presume that by using as isolated parameter of complexity, the dimensions of the hernia may be insufficient to predict the need for a CST [10, 20, 23], and other factors related to the hernia or the patients should be considered. Adequate knowledge of the degree of complexity is important to avoid the risk of over-treating low-complex hernias using components separation. Our results suggest that the classification of complexity of Slater et al. [16] is more useful to predict accurately the need for a CST than the diameter of the defect.

Most of the operations were elective (96.9%), but the operations performed as emergencies presented a high proportion of complications (51% *vs*. 36%; *P* = 0.035), mortality (12.8% *vs*. 0.8%; *P* = 0.014), and recurrences (29.4 *vs.* 9.4; *P* = 0.007), and a 51.7% *vs.* 20.2% (*P* < 0.0001) of the operations were performed in a non-specialized unit. When the operations were performed in an AWU, there were fewer complications (66.7% vs. 28.6%; *P* = 0.04). These data confirm the risks of urgent IH surgery [24] and support the hypothesis that these techniques should be performed, if possible, by specialized surgeons [13] or should be avoided, particularly in higher risk situations, such as emergencies.

Mesh was used in 99% of the operations, as it has been shown to reduce the recurrence rates [5–7]. A CST without mesh was strongly related to recurrences (OR 10.15); this result, in our opinion, suggests that a non-mesh repair should be avoided in these patients, and if the conditions of surgery preclude the use of mesh, as in emergencies, CST should be postponed.

A high rate of complications, as in a previous series [20, 26, 27], was observed. In a previous multivariate analysis of the registry [12], CST itself was a risk factor for complications. Overall complications were similar in ACS and PCS techniques. However, the ACS technique had a higher frequency of skin necrosis (related to the technical characteristics) and wound infections. Most of the other risk factors that emerged from our analysis have been previously reported [16, 20–22]. Interestingly, most of them have been compiled by the complexity classification proposed by Slater et al. [16], and in our series, higher complexity was correlated with a higher rate of complications. Our data seem to validate this system of classification to predict the development of complications in terms of the complexity grade.

The main factor related to less recurrences in the multivariate analysis was an operation performed in an AWU, probably due to better selection of patients and technique, for surgeons with a high volume of complex cases and specially involved in abdominal wall reconstruction. The lack of correlation between the BMI and recurrence is noteworthy, since other authors have found an association [16, 21, 22]. An important appreciation is that age did not influence recurrences; thus, performing CST had a similar safety profile in accordance with previous reports [28].

### Strengths and limitations

In our opinion, this study provides a retrospective and comprehensive review of patients undergoing IH repair using CST, and all patients’ treatment and follow-up were performed prospectively and registered in the largest database of IH in Spain. The registry itself, as previously shown by registries from other countries [29–31], confirms its usefulness for the collection and analysis of data that can be applied to the treatment of IHs.

Nevertheless, we also acknowledge some weaknesses in our study. First, analysis and patient selection relied exclusively on the EVEREG database, with the inability to study the entire patient data recorded due to the lack of basic elements for analysis or errors in their compilation in some cases. Furthermore, EVEREG is just a sample of reality, because our country does not have a universal registry like other countries, which may lead to biases related to the type of patients and the hospitals that treat them and the data analyzed has not been audited. To improve this, we are involved in a project to audit the data on EVEREG registry (Clinical Trials.gov ID: NCT03899012). Other potential problems are that many patients were excluded or lost to follow-up owing to lack of a complete medical record favoring patients that were more likely to follow-up, and the patient populations might have intrinsic differences and factors predisposing to complex IH that we are not aware of. The low percentatge of long-term follow-up is an issue of our Registry due to its voluntary nature and the lack of incentives. The implementation of reward mechanisms and the involvement of government institutions is necessary to improve this aspect. Finally, our research, has the same limitations in the interpretation of its results from other studies based on hernia registries [32].

By contrast, patients operated with minimally invasive surgery were not included. A previous study shows only a 11.7% of all types of repairs with laparoscopy [11]. Thus, we need a national effort to increase the widespread of minimally invasive techniques to achieve better results. In the future, we must determine if laparoscopy and robotics contributes to reducing the number of complications and recurrences as previously reported [33]. However, minimally invasive repair is very demanding and requires specialized units and surgeons for both laparoscopy and AWR.

## Conclusions

AWR continues to be a challenge. While ACS and PCS are effective methods for managing IH, they are complex surgical techniques with high frequency of complications. Elective mesh repair and treatment in an AWU have better recurrence outcomes, reinforcing the real need to improve the status of our patients, create specific abdominal wall surgery units to treat complex hernias, and perform this skilled technique by dedicated surgeons whenever possible.

## Data Availability

The datasets analyzed during the current study are not publicly available due to contracts with the hospitals providing to the database.
